# *Scube3* Is Expressed in Multiple Tissues during Development but Is Dispensable for Embryonic Survival in the Mouse

**DOI:** 10.1371/journal.pone.0055274

**Published:** 2013-01-29

**Authors:** Guilherme M. Xavier, Leonidas Panousopoulos, Martyn T. Cobourne

**Affiliations:** 1 Department of Craniofacial Development and Stem Cell Biology, King's College London Dental Institute, London, United Kingdom; 2 Department of Orthodonticsm, King's College London Dental Institute, London, United Kingdom; Ecole Normale Supérieure de Lyon, France

## Abstract

The vertebrate Scube family consists of three independent members Scube1-3; which encode secreted cell surface-associated membrane glycoproteins that share a domain organization of at least five recognizable motifs and the ability to both homo- and heterodimerize. There is recent biochemical evidence to suggest that Scube2 is directly involved in Hedgehog signaling, acting co-operatively with Dispatched to mediate the release in soluble form of cholesterol and palmitate-modified Hedgehog ligand during long-range activity. Indeed, in the zebrafish myotome, all three Scube proteins can subtly promote Hedgehog signal transduction in a non-cell autonomous manner. In order to further investigate the role of Scube genes during development, we have generated mice with targeted inactivation of *Scube3*. Despite a dynamic developmental expression pattern, with transcripts present in neuroectoderm, endoderm and endochondral tissues, particularly within the craniofacial region; an absence of Scube3 function results in no overt embryonic phenotype in the mouse. Mutant mice are born at expected Mendelian ratios, are both viable and fertile, and seemingly retain normal Hedgehog signaling activity in craniofacial tissues. These findings suggest that in the mouse, Scube3 is dispensable for normal development; however, they do not exclude the possibility of a co-operative role for Scube3 with other Scube members during embryogenesis or a potential role in adult tissue homeostasis over the long-term.

## Introduction

The Scube (Signal peptide CUB EGF-like domain-containing protein) family consists of three independent members evolutionarily conserved from zebrafish to humans [1], [2], [3], [4], [5], [6]. These genes encode secreted and cell surface-associated proteins that share a domain organization of at least five recognizable motifs, including an amino-terminal signal peptide sequence, multiple EGF (epidermal growth factor-like) repeat domains, a large spacer region containing multiple N-linked glycosylation sites followed by three repeated stretches of six-cysteine residues and a C-terminal CUB (Complement subcomponents C1r/C1s, EGF-related sea urchin protein, bone morphogenetic protein 1) domain [1].

Collectively, *Scube1-3* demonstrate dynamic patterns of expression in the vertebrate embryo, which are both reciprocal and complementary to each other, with transcripts predominating in the notocord, central nervous system and somites from the earliest stages of development [1], [2], [3], [4], [5], [7], [8]. There is also evidence that these genes are relevant in a developmental context. Targeted deletion of *Scube1* in mice results in early postnatal lethality associated with significant craniofacial defects, including midbrain neural overgrowth, exencephaly and loss of the cranial vault [9]. In zebrafish, mutations in *Scube2* have been identified in the *you* mutant, characterized by abnormal somite morphology and reduced numbers of both muscle pioneer cells and slow twitch populations within the myotome. These defects are secondary to a loss of long-range Hedgehog signaling in this region, with Scube2 acting in a non-cell autonomous manner [5], [7], [8]. More recently, biochemical evidence from cultured cell assays has emerged to suggest that the mode-of-action underlying Scube2 function is to enhance the secretion and solubility of Sonic hedgehog (Shh), synergizing with Dispatched to facilitate the release of lipid-modified forms of this ligand during long-range signaling [10], [11]. Human *SCUBE3* was originally identified following transcriptional profiling of vascular endothelial cells, demonstrating significant enrichment in primary osteoblasts [6]. In the mouse, *Scube3* is expressed in ectodermal, endodermal and mesodermal-derivatives [3] with transgenic over-expression providing evidence of involvement in the maintenance of myocyte integrity and/or growth during compensatory responses to myocardial stress [12]. Interestingly, SCUBE3 can also complex with TGFβ1 through its carboxy and/or amino-terminal domain, significantly promoting TGFβ1-induced transcriptional activation in HepG2 cells [12].

We have previously investigated *Scube3^tm1Dge/H^* mice, which express a truncated form of Scube3 containing only part of the spacer region and the CUB domain [13]. However, these mice are phenotypically normal, which is consistent with findings in cell culture that a Scube2ΔEGF construct containing only the spacer region, cysteine-rich domains and CUB domain retains functionality [10]. Indeed, at least for Scube2, secretion is mediated by the spacer region C-terminal to the EGF repeats and the cysteine-rich domain, whilst the CUB domain is required for interaction with and release of Shh [14], [15]. In an attempt to further elucidate the role of Scube genes during development, we have generated the first murine model for a loss of *Scube3* function. However, inactivation of *Scube3* in the mouse led to no overt developmental anomalies, suggesting that *Scube3* is not essential for gross embryonic development and survival.

## Results and Discussion

### *Scube3* expression in the developing craniofacial region

**Figure 1:**
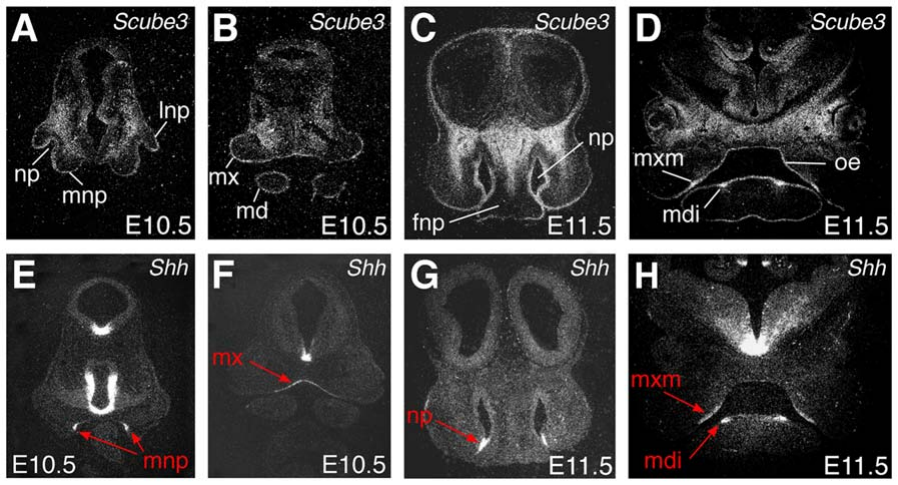
Expression of *Scube3* and *Shh* in the early craniofacial region. Radioactive *in situ* hybridization on frontal sections. (A–D) *Scube3* expression. (A, B) At E10.5, expression is seen primarily in early ectoderm of the facial processes with transcripts also dispersed within the underlying mesenchyme; (C, D) At E11.5, expression is seen throughout the oral ectoderm, including early thickenings of the developing tooth germs, and in the craniofacial mesenchyme. (E–H) *Shh* expression. Regions of overlap between *Shh* and *Scube3* transcription are indicated by red arrows and include: (E, F) At E10.5, ectoderm of the medial nasal and maxillary processes in the midline; (G, H) At E11.5, ectoderm at the base of the nasal pits and early ectoderm of the developing teeth. fnp, fronto-nasal process; lnp, lateral nasal process; md, mandibular process; mdi, mandibular incisor tooth germs; mnp, medial nasal process; mx, maxillary process; mxm, maxillary molar tooth germs; np, nasal pit; oe, oral ectoderm.

**Figure 2:**
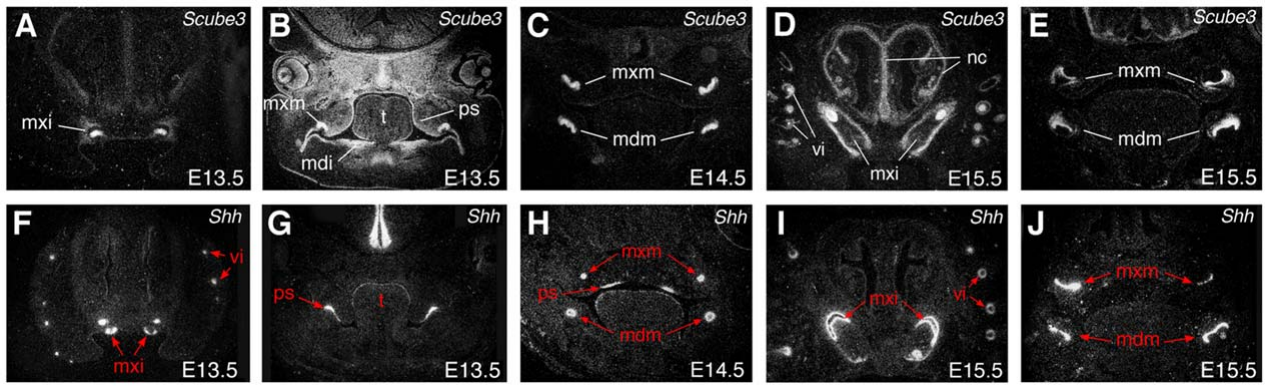
Expression of *Scube3* and *Shh* at later stages of craniofacial development. Radioactive *in situ* hybridization on frontal sections. (A–E) *Scube3* expression. (A, B) At E13.5, expression is seen in ectoderm of the bud stage tooth buds, early palatal shelves and tongue; (C) At E14.5, expression is intense in the cap stage tooth germ; (D, E) At E15.5, strong expression continues in ectodermal tissues of the developing teeth, the vibrissae and cartilaginous condensations of the nasal cavity. (F–J) *Shh* expression. Regions of overlap are between *Shh* and *Scube3* are indicated by red arrows and include: (F, G) At E13.5, ectoderm of the early tooth buds and palatal shelves; (H) At E14.5, cells of the enamel knot within the cap stage tooth germs; (I–J) At E15.5, within the internal enamel epithelium of the bell stage tooth germs and the vibrissae. mdi, mandibular incisor tooth germs; mdm, mandibular molar tooth germs; mxi, maxillary incisor tooth germ; mxm, maxillary molar tooth germs; nc, nasal capsule; ps, palatal shelf; t, tongue; vi, vibrissae.

We have previously demonstrated strong expression of *Scube3* in neuroectoderm of the developing mouse embryo from E8.5, with transcripts localizing to other tissues during later development, including CNS, endoderm and endochondral condensations associated with the early skeleton [3]. In the early craniofacial region, *Scube3* has a dynamic pattern of expression, being identifiable in ectoderm of the facial processes and oral cavity, including the maxillary and mandibular primordia (Fig. 1A–D). Expression is also seen at later stages, in both the tooth germs and vibrissae, organs that are known to develop through coordinated signaling between ectoderm and mesenchyme, and within early cartilaginous regions of the skull, including the nasal cavity (Fig. 2A–E). Interestingly, given the known associations between Scube2 and Hedgehog signaling, during all these early stages of craniofacial development some overlap in expression domains exists between *Scube3* and *Shh*, particularly in ectodermal tissues of the developing face, the teeth and vibrissa (Fig. 1E–H; Fig. 2F–J, red arrows). These overlapping domains are consistent with a potential role for Scube3 as a facilitator of Shh signaling during early facial development.

### Structure and organization of murine *Scube3*

**Figure 3:**
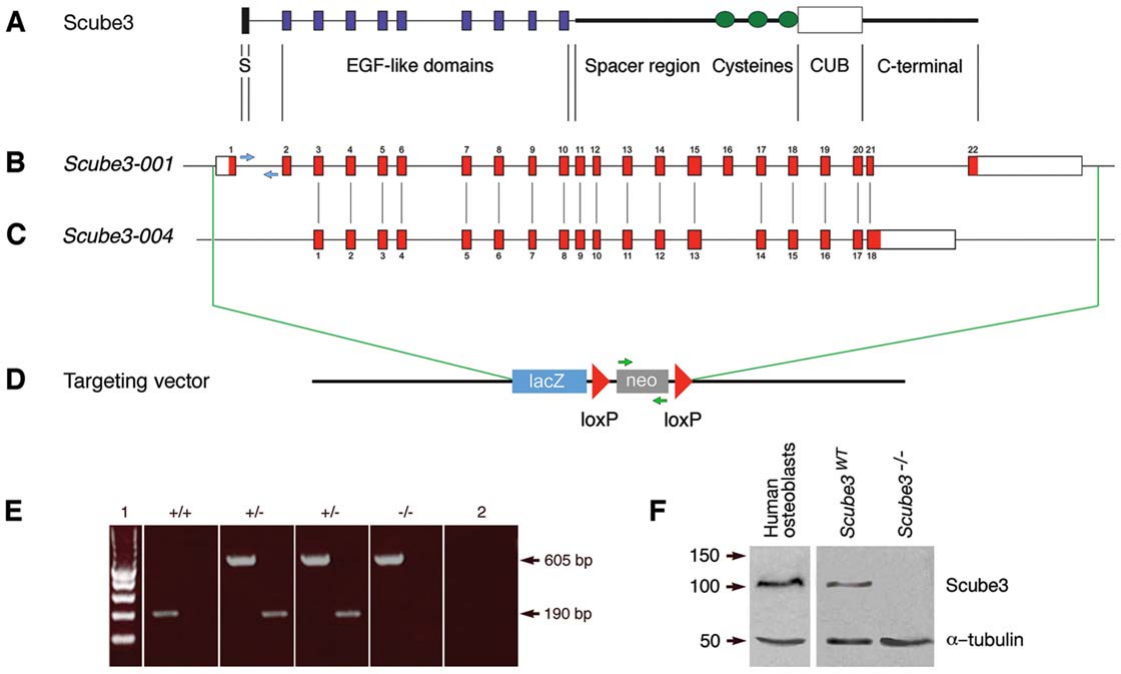
Targeted disruption of the *Scube3* gene in mice. (A) Domain organization of Scube3 encoded by the *Scube3-001* splice variant. This protein consists of a signal peptide sequence (S), nine EGF-like repeat domains, a spacer region containing three cysteine-rich repeats, a CUB domain and C-terminal sequence; (B) The murine *Scube3*-*001* splice variant incorporates 6699 base pairs and contains 22 exons. Exon 1 encodes the signal peptide sequence, exons 2–10 encode the nine EGF-like domains, exons 11–18 encode the spacer region and cysteine residues in proximity to the CUB domain, exons 19 and 20 encode the CUB domain itself and exons 21–22 encode the amino acid sequence present at the C-terminal region of the protein, downstream of the CUB domain; (C) The *Scube3-004* splice variant incorporates 4103 base pairs and contains 18 exons. The splice variants are aligned to each other, meaning that exon 1 of *Scube-004* corresponds to exon 3 of *Scube-001* and so forth. This is interrupted by exon skipping at exon 16 in *Scube-001*, such that intron 13–14 of *Scube-004* corresponds to intron 15–16, exon 16 and intron 16–17 of *Scube3-001* (a total region of 714 base pairs). Exon 18 of *Scube3-004*, which corresponds in part, to exon 21 of *Scube-001* is also modified by an alternative 5′ splice site, incorporating 1684 ‘extra’ base pairs from the adjacent intronic sequence (intron 21–22 of *Scube-001*). Red boxes represent exons, white boxes represent untranslated regions of exon 1 and 22; (D) The targeting vector contains a ZEN-Ub1 cassette consisting of a *lacZ-p(A)* reporter and *hUbCpro-neo-p(A)* selectable marker flanked by *loxP* sites, which replaces the whole *Scube3* coding region following homologous recombination in ES cells (A–D are not to scale); (E) PCR analysis of DNA extracted from tail samples using wild type and mutant pairs of primers for wild type and mutant alleles, respectively. The wild type set of primers recognize a 25 base pair intronic sequence situated between exon 1 and 2 (blue arrows) and amplify a DNA fragment of 190 base pairs; whilst the mutant pair of primers amplify a 605 base pair DNA fragment within the Neo cassette (green arrows). For each sample, two separate PCR reactions were run (1) 100 base pair DNA ladder; (2) Negative control; (F) Western blot analysis reveals an absence of Scube3 in protein lysate derived from *Scube3^−/−^* embryos. Protein lysates derived from human osteoblasts and *Scube3^WT^* mouse embryos were used as positive controls. α-tubulin was used as a loading control. As predicted, the Scube3 protein detected in human osteoblasts and wild type embryos was approximately 110 kDa in size.

Mouse *Scube3* encodes a large secreted glycoprotein composed of an amino-terminal signal peptide, multiple EGF-like domains, a large spacer region rich in N-linked glycosylation sites and a C-terminal CUB domain (Fig. 3A) and is located on chromosome 17 at location 28142316–28174852, extending over 32.5 kilobases [Vertebrate Genome Annotation (VEGA) database: OTTMUSG00000021032]. Murine S*cube3* has five splice variants (*Scube3 001–005*); however, three of these do not have an open reading frame and do not encode protein (*Scube3-002; -003; -005*). The remaining two encode different proteins; the *Scube3-001* transcript (OTTMUST00000049724) has 22 exons, 6699 base pairs and 993 amino acid residues (Fig. 3B), whilst *Scube3-004* (ID OTTMUST00000049727) has 18 exons, 4103 base pairs and 828 amino acids (Fig. 3C). Currently, little is known about the biological relevance of either protein.

### Generation of *Scube3^tm1(KOMP)Vlcg/tm1(KOMP)Vlcg^* mice

The overall expression pattern associated with *Scube3* was suggestive of a potential role for the encoded protein during embryonic development. In order to investigate this further, we have generated mice lacking *Scube3* function throughout development. Targeting of the *Scube3* locus in ES cells and subsequent germline transmission was carried out at the University of California, Davis within the auspices of the National Institute of Health Research (NIHR) Knockout Mouse Project (KOMP). Within this strategy, the targeted *Scube3* allele was completely replaced by a ZEN-Ub1 [*lacZ-(pA)-hUbiPro-neo-p(A)*] promoter-driven expression-selection cassette to produce a mutant (*tm1(KOMP)Vlcg*) allele (Fig. 3D).

### *Scube3^+/tm1(KOMP)Vlcg^* embryos reproduce normal *Scube3* expression

**Figure 4:**
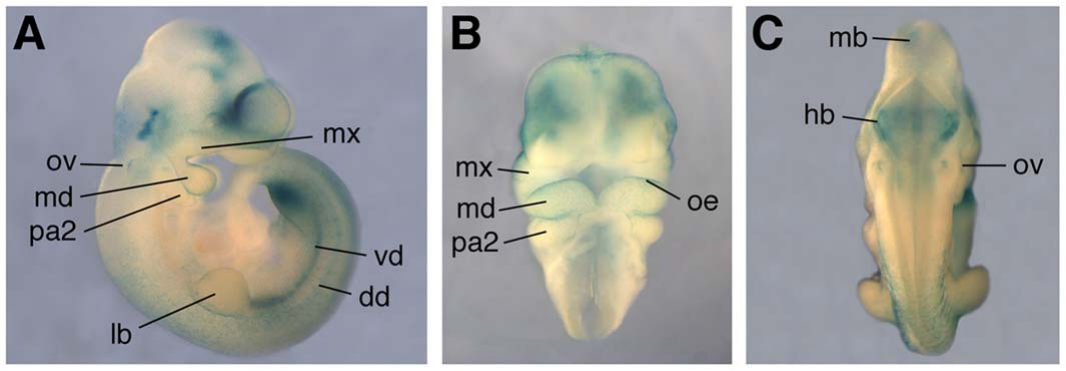
*Scube3^+/tm1(KOMP)Vlcg^* reporter gene activity at E10.5 visualized by β-galactosidase staining. (A) Lateral view; (B) Frontal view (of the craniofacial region); (C) Posterior view. Note reporter expression in the central nervous system and ectoderm of the facial processes, otic vesicle and limb buds. Strong posterior-restricted expression is also seen in the limb bud mesenchyme [13] (only visible in the hindlimb of this sample). dd, dorsal dermomyotome; flb, forelimb bud; hb, hindbrain; hlb, hindlimb budlb, limb bud; mb, midbrain; md, mandibular process; mx, maxillary process; oe, oral ectoderm; ov, otic vesicle; pa2, pharyngeal arch 2; rh, rhombencephalon; vd, ventral dermomyotome.

Heterozygous mice carrying the *tm1(KOMP)Vlcg* allele (*Scube3^+/−^*) were maintained at King's College London on a C57/Bl6 background and genotyped by PCR (Fig. 3E). The presence of *lacZ* in the mutant allele allowed us to investigate and verify the domains of *Scube3* activity in *Scube3^+^* ^/*tm1****(KOMP)Vlcg***^ embryos. Reporter activity in these mice recapitulated the embryonic expression domains previously reported for *Scube3* [3] and closely correlated with those identified in a *Scube3^tm1Dge/H^* reporter line [13]. In particular, at E10.5 *Scube3* reporter activity was clearly visible in the hindbrain, otic vesicle, facial ectoderm and pharyngeal arch system, limb bud and ventral dermomyotome (Fig. 4A–C).

### *Scube3^tm1(KOMP)Vlcg/tm1(KOMP)Vlcg^* mice are viable and fertile

*Scube3^+/−^* mice were crossed to generate *Scube3^tm1^* ^***(KOMP)Vlcg****/tm1****(KOMP)Vlcg***^ (*Scube3^−/−^*) mice homozygous for the deleted allele. *Scube3^−/−^* mice were born at expected Mendelian ratios but remained viable and fertile, demonstrating seemingly normal embryonic development and being grossly indistinguishable from wild type (*Scube3^WT^*) or *Scube3^+/−^* litter mates. These findings prompted us to confirm an absence of Scube3 protein in the mutants using Western blot analysis on protein lysates derived from both *Scube3^WT^* and *Scube3^−/−^* mice. As predicted from the targeting strategy, in contrast to *Scube3^WT^* mice, no Scube3 protein was detected in *Scube3^−/−^* animals (see Fig. 3F). These findings confirmed an absence of functional Scube3 protein in the mutant and demonstrated that Scube3 function is not essential for gross murine development or survival. However, we sought to further investigate the phenotype of these mice, focusing in more detail on those structures that demonstrated significant expression of *Scube3* during embryonic development.

**Figure 5:**
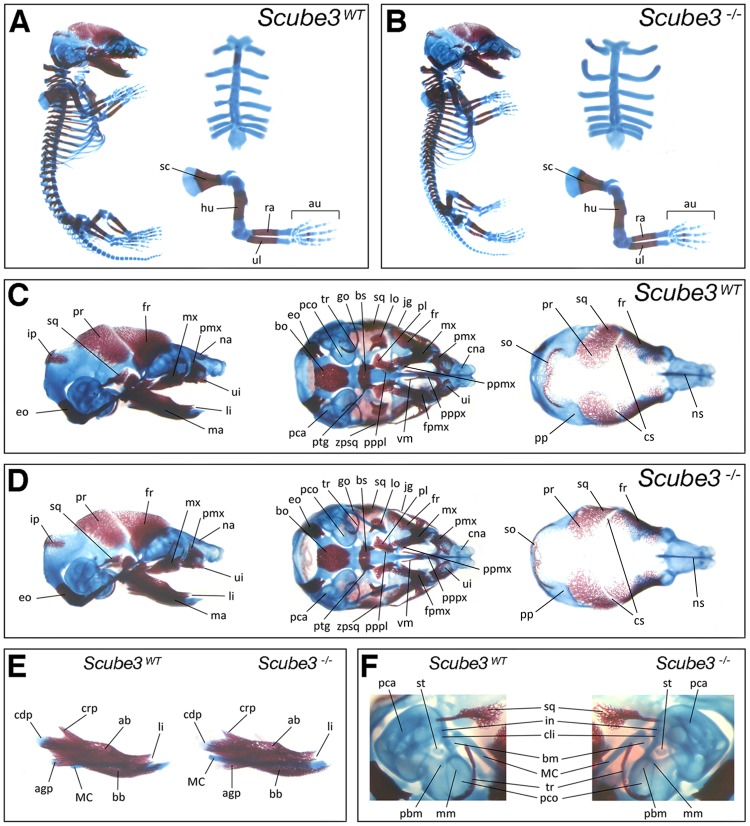
Skeletal morphogenesis in wild type and *Scube3^−/−^* mice. Comparison of E17.5 wild type and *Scube3^−/−^* skeletal structures differentially stained for bone (alizarin red) and cartilage (alcian blue). (A, B) Gross appearance of the skeleton, including sternum and forelimb; (C, D) Skull, including lateral view (norma lateralis, left panels), cranial base (norma basalis, middle panels; note, the mandible has been removed), cranial vault (norma verticalis, right panels; note, the cranial base and nasal bones have been removed); (E) Mandible (lateral view); (F) Middle ear skeletal elements (right ear in wild type; left ear in mutant). ab, alveolar bone of mandible; agp, angular process of mandible; au, autopod; bb, basal bone of mandible; bm, body of malleus; bo, basioccipital; bs, basisphenoid; cdp, condylar process of mandible; cli, crus longus of incus; cna, cupola nasi anterior; crp, coronoid process of mandible; cs, coronal suture; eo, exoccipital; fpmx, frontal process of maxilla; fr, frontal; go, gonial; hu, humerus; in, incus; ip, interparietal; jg, jugal; li, lower incisor; lo, lamina obturans; ma, mandible; MC, Meckel's cartilage; mm, manubrium of malleus; mx, maxilla; na, nasal; ns, nasal septum; pbm, processus brevis of malleus; pca, pars canalicularis; pbm, processus brevis of malleus; pco, pars cochlearis; pl, palatine; pmx, premaxilla; pp, parietal plate; ppmx, palatine process of maxilla; pppl, palatine process of palatine; pppx, palatal process of premaxilla; pr, parietal; ptg, pterygoid; ra, radius; sc, scapula; so, supraoccipital; sq, squamosal; st, stapes; tr, tympanic ring; ui, upper incisor; ul, ulna; vm, vomer; zpsq, zygomatic process squamosal.

The developmental expression pattern associated with *Scube3* had been predictive of a potential role in development of axial and craniofacial skeletal elements. We therefore investigated this possibility by comparing skeletal preparations of E17.5 *Scube3^WT^* and *Scube3^−/−^* mice. Gross organization of the axial skeleton was normal in the mutant, with no obvious differences in either size or overall morphology between genotypes (Fig. 5A, B). In particular, analysis of the craniofacial region demonstrated normal dermatocranial and chondrocranial elements within the mutant skull (Fig. 5C–F). Specifically, the facial skeleton was intact and normally organized, with skeletal elements within both the primary and secondary palates and mandible all normally formed in the mutant. In addition, organization of middle and inner ear structures was grossly normal, including the three middle ear ossicles and both the pars canalicularis and cochlearis of the inner ear (Fig. 5F).

**Figure 6:**
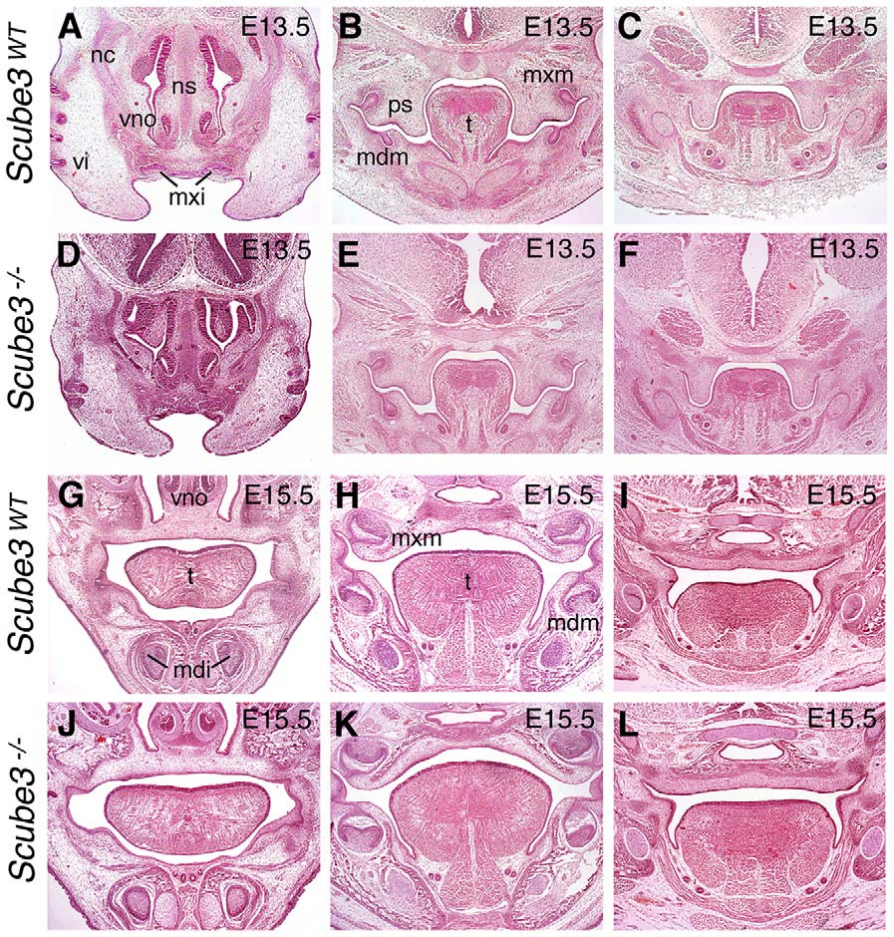
Craniofacial development in wild type and *Scube3^−/−^* mice. Histological comparison of the developing craniofacial region in wild type and *Scube3^−/−^* mice at E13.5 and E15.5. (A–C) E13.5 wild type; (D–F) E13.5 mutant; (G–I) E15.5 wild type; (J–L) E15.5 mutant. All sections are orientated in the frontal plane and stained with Haematoxylin and Eosin. Orientation is through the primary palate (left panels), central region (middle panels) and posterior region (right panels) of the secondary palate. mdi, mandibular incisor tooth germs; mdm, mandibular molar tooth germs; mxi, maxillary incisor tooth germs; mxm, maxillary molar tooth germs; nc, nasal cavity; ns, nasal septum; ps, palatal shelf; t, tongue; vi, vibrissae; vno, vomeronasal organ.

Histological analysis of the craniofacial tissues at E13.5–15.5 further demonstrated that gross development of this region was normal in the mutant, when compared to wild type (Fig. 6A–L). In particular, the developing facial processes, nasal cavity, palate and dentition all appeared normal in the mutant.

### Early tooth development proceeds normally in *Scube3^−/−^* mice

**Figure 7:**
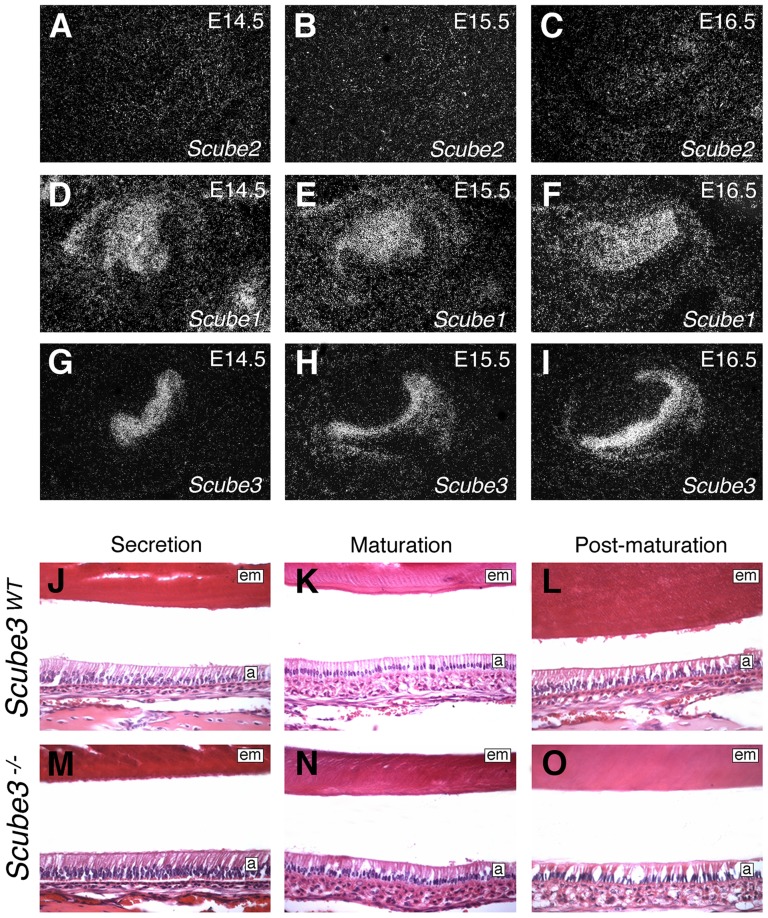
Scube gene function in tooth development. (A–I) *Scube* gene expression in the developing first molar at E14.5–16.5 assayed by *in situ* hybridization on frontal sections. (A–C) *Scube2*; (D–F) *Scube1*; (G–I) *Scube3*. (J–O) Ultra-structural view of incisor tooth development in wild type and *Scube3^−/−^* mice at P18. In the incisor, amelogenesis commences apically and proceeds in an coronal direction, demonstrating (J, M) Secretion; (K, N) Maturation; (L, O), Post-maturation. These different stages of amelogenesis all show a characteristically normal morphology in wild-type and *Scube3^−/−^* mice. a, ameloblasts; em, enamel matrix.

In zebrafish, current evidence suggests that Scube2 is the dominant member of this family in facilitating long-range Hedgehog signaling, at least within the developing myotome [4]. Whilst *Scube2* expression does extend beyond the myotome in both fish and mouse embryos [4], [16], expression in the developing mouse tooth is negligible, only being present above background levels from around E16.5 within the mesenchyme (Fig. 7A–C). In contrast, both *Scube1* and *Scube3* are strongly expressed during murine odontogenesis, in both incisor and molar teeth, demonstrating reciprocal patterns within mesenchymal and ectodermal compartments, respectively (Fig. 7D–F; G–I) [3], [13], [17]. In particular, *Scube3* is expressed in the primary epithelial thickening during the initiation of tooth development, localizing progressively to the basal tooth bud, internal enamel epithelium and then enamel-secreting ameloblasts of the bell stage tooth germ [3]. Collectively, these observations suggest that *Scube3* might potentially have a more significant role in the tooth compared with other regions of the embryo. Given their complementary expression domains, Scube3 could potentially play a role in mediating the distribution of Shh at long-range during odonotogenesis [18]. We therefore compared gross morphology of the developing incisor and molar tooth germs between wild type and *Scube3^−/−^* mice using histology at the bud and late cap stages. However, tooth germs were morphologically indistinguishable between wild type and mutant at both these stages of development (see Fig. 5A, B; D, E and G, H; J, K). Given the presence of *Scube3* expression in differentiated ameloblasts at later stages of development (data not shown) we further analyzed this cell population in mandibular incisor teeth derived from post-natal day (P) 18 wild type and *Scube3^−/−^* mice (Fig. 7J–O). In wild type mice, ameloblasts pass through a series of distinct morphological changes during their life-cycle, which are all visible in the mature incisor tooth and extend from apical to coronal regions, as these cells initially secrete the enamel matrix, enter a transitional phase and then subsequently increase mineral content during maturation. In particular, these cells decrease in height, increase in width and lose their characteristic Tomes' process, becoming almost cuboidal during post-maturation [19] (Fig. 7J–L). In *Scube3^−/−^* incisors, this pattern of morphological change was maintained in the ameloblasts, with evidence of both a normal life cycle and enamel matrix secretion (Fig. 7M–O). Therefore, despite the presence of *Scube3* alone in the ectodermal compartment of the developing tooth, this gene would not appear to be essential on a gross level for normal odontogenesis.

### Shh signaling is normal in the craniofacial region of *Scube3^−/−^* embryros

**Figure 8:**
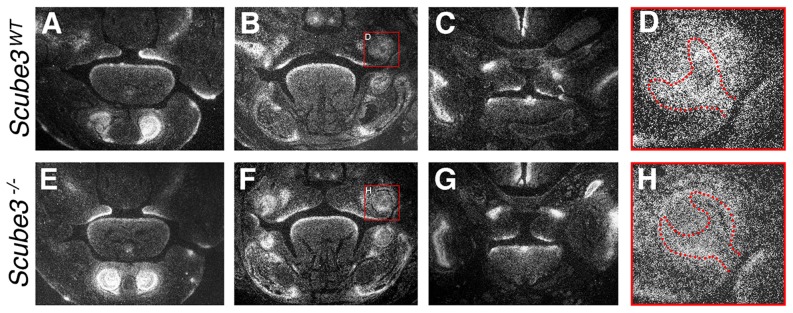
Hedgehog signaling in wild type and *Scube3^−/−^* mice. Comparison of *Ptch1* expression in the developing craniofacial region of wild type and *Scube3^−/−^* mice at E14.5 assayed by *in situ* hybridization on frontal sections. (A–D) Wild type; (G–H) *Scube3^−/−^*. Orientation is through the primary palate (A, E), central region (B, F) and posterior region (C, G) of the secondary palate. Red square in B, F highlights *Ptch1* expression in the cap stage maxillary molar teeth shown in D, H (red hatched line outlines the enamel organ).

In the zebrafish embryo, Scube gene function is required for normal Hedgehog signal transduction, with partial redundancy existing between the three members of this family. Loss of *Scube2* results in the *you* mutant phenotype, whilst morpholino knockdown of *Scube1* or *Scube3* on a wild type background, either alone or together, results in essentially normal development. However, in a *Scube2* mutant background, knockdown of *Scube1* or *Scube3* produces enhancement of the muscle fibre type phenotype associated with the *you* mutant, with knockdown of all Scube gene function associated with an increased severity of the phenotype and more significant loss of Hedgehog signaling [4]. We therefore assayed levels of Hedgehog signaling in the craniofacial region of *Scube3^−/−^* mice using *in situ* hybridization for *Ptch1*, a known downstream transcriptional target of this pathway. The absence of any significant phenotype in *Scube3^−/−^* mice was accompanied by seemingly normal levels of *Ptch1* in the craniofacial region, including the neural tube, tooth and palatal shelves (Fig. 8A–H molar tooth germs highlighted).

Although widely expressed in the mouse embryo [1], [2], [3], [13], [16], [17] there is currently only limited data relating to the function of Scube genes during mouse development. In mice with targeted disruption in *Scube1*, there is normal limb and skeletal development but acrania, associated with severe disruption of the flat bones within the cranial vault and exencephaly, possibly secondary to a role in antagonizing BMP signaling [9]. A mouse model for a loss-of-function in *Scube2* has not been described, but during zebrafish development [4], [5], [7] and in cell culture [10], [11], current evidence suggests a role for this protein during Shh signaling, primarily by acting with Dispatched in facilitating the release of cholesterol-modified Shh from signaling cells. However, the phenotype associated with loss of *Scube2* function in zebrafish is subtle, producing a loss of Hedgehog signaling and slow myosin heavy chain muscle fibre formation in the posterior somite region [4]. Moreover, although Scube2 seems to predominate, there is clearly a complex and partially redundant relationship between all Scube proteins during this process, possibly reflecting combinatorial homo- and heterodimerization between members in this region [4]. How Scube proteins might subtly influence signaling in other regions of Hedgehog co-expression and what the significance of Scube expression in regions devoid of Hedgehog signaling is not currently understood, although an ability to antagonize BMP signaling might be related to their expression in dorsal regions of the developing CNS.

A further interesting feature associated with Scube genes is the reported expression of all family members in adult human tissues, including *SCUBE1* in endothelium and platelets [15], [20], *SCUBE2* in smooth muscle cells and fibroblasts [15] and *SCUBE3* in primary osteoblasts [6]. Interestingly, transgenic mice over-expressing *Scube3* through a *Colla1* promoter have no skeletal phenotype and are developmentally normal up to 2 months of age. However, *Scube3* is ventricle-enriched in these mice and at 8 months they begin to demonstrate abnormalities in their ECG pattern, secondary to ventricular hypertrophy [12]. We cannot discount any long-term pathological effects associated with loss of Scube3 function in the mouse and this is the subject of our current ongoing investigations.

### Conclusions

*Scube3* encodes a secreted cell surface-associated protein that exhibits a dynamic expression pattern in the developing mouse embryo, particularly in ectodermal-derivatives of the early craniofacial region. Here, we have generated mice lacking *Scube3* function and find that they remain viable and fertile, demonstrating no overt embryonic phenotype. Therefore, we conclude that on a C57/Bl6 background at least, *Scube3* is not essential for gross embryonic development and survival. These findings might indicate redundancy with other Scube family members during development and do not discount a potential role in adult tissue homeostasis over the long-term.

## Materials and Methods

### Ethics statement

Animal work was carried out within strict ethical guidelines under the appropriate United Kingdom Government Home Office License (70/5996).

### Targeting construct, generation and genotyping of *Scube 3^−/−^* mice

The mouse line analyzed in this study carried a targeted disruption of *Scube3* generated by VelociGene® (Regeneron Pharmaceuticals Inc) high throughput gene targeting, as part of the National Institutes of Health (NIH) Knockout Mouse Project. Specifically, in this strategy *Scube3* is completely replaced by a ZEN-Ub1 promoter-driven expression selection cassette within a BacVec targeting vector (Velocigene ID: 12567). ZEN-Ub1 contains a *lacZ* β-galactosidase coding sequence and a neomycin-resistance gene downstream of a human *UBIQUITIN C* gene [*lacZ-(pA)-hUbiPro-neo-p(A)*]. The *hUbiPro-neo-p(A)* sequence is flanked by *LoxP* sites but there is no conditional targeting potential. The targeting vector was designed to cause both a deletion of the *Scube3* target gene sequence and the insertion of a neomycin-resistance selectable marker, such that the reading frame of the protein coding sequence is interrupted. The deletion size consisted of 28837 base pairs, which completely covers the *Scube3* locus. The identity of the gene target and integrity of the vector were confirmed by DNA sequence analysis of the construct outward from the cassette insertion site (data not shown).

Electroporation of the ZEN-Ub1 cassette into ES cells, selection for neomycin-resistance, identification of cell lines undergoing homologous recombination of ZEN-Ub1 into the *Scube3* locus, injection of relevent ES cells into blastocysts, implantation of embryos, breeding of chimeric male offspring to C57/BL6 females and screening of F1 animals was all carried out by standard methods under the auspices of KOMP (data not shown). *Scube3^+/−^* mice were imported from KOMP and a colony established at King's College London. The *Scube3^+/−^* line was maintained on a C57/BL6 background and routinely genotyped using PCR analysis of tail or ear DNA. One primer set (WT1: 5′-AACCAACTCACGCCTAAAAGTGTCG-3′; WT2: 5′-GGCCCTAAATCAAATCGTACCCAGC-3′) amplified a 190 base pair product from the wild type *Scube3* allele; whilst another primer set (M1: 5′-GCAGCCTCTGTTCCACATACACTTCA-3′; M2: 5′-ACTTGTAAACCCTATGGAGTCAGCCC-3′) amplified a 605 base pair product from the mutant *Scube3* allele in two distinct reactions.

### Sample collection

*Scube3^+/−^* mice were time-mated and pregnant females sacrificed with cervical dislocation. Matings were set up such that noon of the day on which vaginal plugs were detected was considered as embryonic day (E) 0.5 and embryos were collected at different stages.

For standard histology, embryos were fixed in 4% (w/v) paraformaldehyde at 4°C overnight, washed in PBS and dehydrated through a graded ethanol series. Following this, they were embedded in paraffin wax, sectioned at 7 µm and prepared, either for haematoxylin and eosin staining or section *in situ* hybridization. Mandibles from 2-month-old mice (3 *Scube3^WT^* and 3 *Scube3^−/−^*) were dissected following cervical dislocation and fixed in neutral buffered formalin at room temperature for 48 h. Following fixation, mandibles were decalcified in 5% EDTA, dehydrated through a graded ethanol series, cleared in chloroform, embedded as hemi-mandibles in paraffin wax, sectioned and stained with haematoxylin and eosin.

### Radioactive *in situ* hybridization

Radioactive *in situ* hybridization was carried out as previously described [13]. Antisense ^35^S-UTP radio-labelled riboprobes were generated from mouse cDNA clones that were gifts from different laboratories: *Scube1* [1]; *Ptch1* [21] and *Gli1* [22]. The *Scube3* cDNA clone has been previously described and is predicted to lack exons 1, 2 and 16 [3]. Dark-field images of sections were photographed using a Zeiss Axioscop microscope (Germany) and imported into Adobe Photoshop CS.

### Western blot analysis

Western blot analysis was carried out as previously described [13]. A mouse polyclonal antibody against human SCUBE3 (Abnova H00222663-A01) and mouse monoclonal antibody (DM1A) against chicken α-Tubulin (Santa Cruz Biotechnology, sc-32293) were used as primary antibodies at dilutions of 1∶2000 and 1∶5000, respectively. A rabbit anti-mouse antibody conjugated with Horseradish Peroxidase (DAKO) was used as the secondary antibody at a dilution of 1∶2000.

### LacZ staining

For LacZ staining embryos were fixed (2% formaldehyde; 0.2% gluteraldehyde; 0.02% Nonidet P-40; 0.01% sodium deoxycholate in PBS) on ice for 1 hour and then stained with 1 mg/ml X-Gal (Gold Bio Technology) in β-galactosidase stain base (5 mM K3Fe(CN)6; 5 mM K4Fe(CN)6; 2 mM MgCl2; 0.02% Nonidet P-40 in PBS) at 37°C overnight. Embryos were then photographed directly in PBS on a background of agarose using a Leica stereomicroscope.

### Skeletal preparation

For differential staining of bone and cartilage, E17.5 mice were fixed overnight in 95% ethanol, skinned and eviscerated. Cartilage staining was carried out by soaking in a solution of 95% ethanol, 20% glacial acetic acid and 0.015% alcian blue 8GX for 24 hours, differentiating for 7 days in 95% ethanol, macerating in 1% KOH for 24 hours and washing overnight under running tap water. Bone staining was carried out with 0.1% aqueous alizarin red supplemented with several drops of 1% KOH to enhance darkness of the red. Samples were washed for 30 minutes under running tap water, decolorized in 20% glycerol in 1% KOH for 1–2 weeks and submerged in increasing concentrations of glycerol in 70% ethanol to a final concentration of 100% glycerol. Skeletal preparations were photographed in 100% glycerol using a Leica stereomicroscope.
