# Correction: *Scube3* Is Expressed in Multiple Tissues during Development but Is Dispensable for Embryonic Survival in the Mouse

**DOI:** 10.1371/annotation/b65bdf5d-4917-4936-9d9d-f217ab2602cd

**Published:** 2013-09-23

**Authors:** Guilherme M. Xavier, Leonidas Panousopoulos, Martyn T. Cobourne

An error in affiliation 2 for author Martyn T. Cobourne was introduced in the preparation of this article for publication. Affiliation 2 should be: Department of Orthodontics, King's College London Dental Institute, London, United Kingdom

